# A whole-health–economy approach to antimicrobial stewardship: Analysis of current models and future direction

**DOI:** 10.1371/journal.pmed.1002774

**Published:** 2019-03-29

**Authors:** Monsey McLeod, Raheelah Ahmad, Nada Atef Shebl, Christianne Micallef, Fiona Sim, Alison Holmes

**Affiliations:** 1 Centre for Medication Safety and Service Quality, Pharmacy Department, Imperial College Healthcare National Health Service (NHS) Trust, London, United Kingdom; 2 National Institute for Health Research Health Protection Research Unit, Healthcare Associated Infections and Antimicrobial Resistance, Imperial College, London in partnership with Public Health England, Hammersmith Hospital, London, United Kingdom; 3 Department of Clinical and Pharmaceutical Sciences, University of Hertfordshire, Hatfield, United Kingdom; 4 Pharmacy Department, Cambridge University Hospitals NHS Foundation Trust, Addenbrooke's Hospital, Cambridge, United Kingdom; 5 Institute for Health Research, University of Bedfordshire, Luton, United Kingdom; 6 NHS England (Midlands & East), United Kingdom

## Abstract

In a Policy Forum, Alison Holmes and colleagues discuss coordinated approaches to antimicrobial stewardship.

**Summary points**

- Antimicrobial stewardship (AMS) strategies are widely implemented in single healthcare sectors and organisations; however, the extent and impact of integrated AMS initiatives across the whole health economy are unknown.
- Assessing degree of integration of AMS across the whole health economy and its impact is essential if we are to achieve a ‘One Health’ approach to addressing antimicrobial resistance (AMR), and therefore we searched systematically for and analysed published examples of integrated AMS initiatives to address this gap.
- Application of a system-level framework to analyse integration of AMS initiatives across and within healthcare sectors shows that integration is emerging but needs strengthening.
- Findings from a small number of evaluations in high-income countries suggest that antimicrobial prescribing and healthcare-associated infections can be reduced using a multisectoral integrated AMS approach.
- More robust research designs to evaluate and understand the impact of multisectoral integrated AMS are needed, particularly with respect to differing health systems in different countries and local organisational contexts.
- Our analysis highlights a number of challenges and ways forward for enhancing the delivery of AMS through an integrated approach.

## Background

It is estimated that around 700,000 people die annually from drug-resistant infections, with experts predicting an alarming possible increase to 10 million deaths each year by 2050 and major future challenges to the way we practice medicine and surgery [1,2]. It was welcome news that tackling antimicrobial resistance (AMR) and infectious diseases along with health system strengthening were featured at the G20 summit (November, 2018), under the wider aim of improving sustainability, and progress towards more coordinated international efforts will be reviewed at the 73rd session of the UN General Assembly (September 2018) [3]; but how are health professionals, managers, and policymakers assuring coordinated efforts within human healthcare? Globally, there has been much emphasis on a ‘One Health’ approach that involves connecting the health of humans, animals, and the environment to tackle AMR [2]. This is driving much-needed antimicrobial stewardship (AMS) activities in animal production sectors [4]. However, we have yet to achieve and establish joined-up approaches within human health. This paper, therefore, focuses on an analysis of multisectoral AMS in human health. AMS remains a cornerstone for addressing AMR with numerous initiatives implemented with varying degrees of success [5,6]. A critical gap we have identified is that approaches have largely focused efforts separately in primary care or secondary care, and have also heavily targeted medical prescribers. In this paper, we propose that policymakers, clinical leaders, and healthcare managers assess and consolidate AMS activities across the whole health economy, and we use a novel, to our knowledge, approach to demonstrate how such an assessment can be made. We present the extent to which existing AMS initiatives are multisectoral or integrated across a whole health economy within individual countries and their impact on antimicrobial-related outcomes. We then highlight some challenges and key considerations for developing and harnessing potential benefits of integrated AMS approaches.

## Need for a whole-health–economy approach

Health systems are required to deliver best outcomes efficiently, facing the challenges of macroeconomic constraints, technology costs, and increasing public need and demand. Consolidating the sometimes disparate programs and initiatives within the health sector is necessary, and integrated models of care across primary, secondary, tertiary, and long-term care can help with coordinated implementation of AMS [7]. Assessment of the degree of integration of AMS across the whole health economy is essential if we are to understand how a ‘One Health’ approach to addressing AMR may be achieved. Much AMS activity has been concentrated in hospital settings, creating a practical but somewhat artificial boundary that neglects bidirectional influences between hospital and community care services. Antimicrobial use in the community is associated with the development of AMR in and outside hospitals [8]. Furthermore, use of accident and emergency departments by ambulatory patients contributes to fragmented care and overuse of antimicrobials [9]. The way people access healthcare has evolved: the availability of blended care and complex patient-care pathways in some countries allows for patient-centred approaches as well as more rational use of services. The availability of antimicrobials without a prescription in some countries and increasing availability of online pharmacies provides an additional challenge for AMS. Fundamentally, AMS is lagging behind the advances made in health service delivery and patient behaviours by remaining sector-based.

## What does integration mean and how can we assess it?

The One Health perspective on integration involves multiple sectors communicating and working together to design and implement programs, policies, legislation, and research to achieve better public health outcomes [2]. In practice, in England, new integrated care models are being developed through 50 selected collaborative organisations that will inform potential redesign of the whole health system, and 25 integrated care pioneer sites to test new and different ways of joining up health and social care services [10]. Elsewhere in Europe, the Dutch Ministry of Health, Welfare, and Sport established nine pioneer sites to integrate clinical and community services with the aim of achieving ‘better healthcare at lower cost’ [11]. In the United States, accountable care organisations—which typically involve multiple physician practices and at least one hospital—have been established to improve the quality of care while lowering costs [12]. However, AMS is not explicit in any of these wider health-system–integration models.

To further complicate matters, there is no standard definition of integration, and a number of integrated care models have been proposed in the literature [13–17] (**S1 Table**). In this analysis, we define and summarise the extent of integration based on the six facets of critical health system function described by Atun and colleagues [16,18] because it provides a practical level of granularity on the concept of intervention integration and is specific to healthcare (**Table 1**). We appreciate that there may be unpublished initiatives. However, as a novel, to our knowledge, analysis of this issue, the focus was on examining evidence of integrated AMS initiatives from the literature so that some measures of impact and associated context can be synthesised. Our aim was to identify practical considerations to support policymakers seeking to develop integrated AMS across the whole health economy. We carried out a systematic search of the literature published between January, 2006 and December, 2018, selected relevant articles using prespecified inclusion criteria, and reviewed evaluative studies (S1 Appendix). This paper describes an analysis based on 16 AMS initiatives from nine high-income countries and one low-middle–income country (**Tables 2 and 3**).

**Table 1:** Critical health system functions and elements of integration adapted from Atun and colleagues [16,18] for AMS initiatives.

Facets of Critical Health System Function	Elements of Integration Adapted for AMS Initiatives
Stewardship and governance	• Regulatory mechanism • Accountability framework
Financing	• Pooling of funds • Provider payment methods • Funding source • Cross-program use of funds
Planning	• Planning
Service delivery	• Human resources for delivery of AMS • Physical infrastructure for laboratory testing
Monitoring and evaluation	• Data collection and recording • Data analysis • Reporting systems • Performance management system
Demand generation	• Financial incentives • Information, education, and communication

Definition of full and partial integration: An element was classed as fully or predominantly integrated across the health system if it was exclusively under the management and control of the wider healthcare system. An element was classed as partially integrated if some but not all cases were managed and controlled both by the wider healthcare system and a specific program-related structure. A dimension was not integrated if it was exclusively under the management and control of a specific program-related structure (which is distinct from the wider healthcare system). **Abbreviations**: AMS, antimicrobial stewardship.

**Table 2:** Impact of 16 integrated AMS initiatives identified.

AMS Initiative	Study Design/Type	Reported Impact	Limitations for Future Work
Australia			
Infection control nurse consultant in residential aged care facilities [19]	Uncontrolled before and after study	Reduction in the use of cephalexin, doxycycline, flucloxacillin, clindamycin, and metronidazole. Rates of infection types remained stable, except respiratory tract infection rates increased at one of the two study sites.	No control group
National multistrategic AMS program for health professionals and the community [20]	Uncontrolled before and after study	Continued decline in total volume of antibiotics prescribed, GPs and pharmacists perceived the campaign assisted in AMS message promotion to patients, improvement in consumer knowledge and attitudes about self-management of infections	Possible impact of other national level campaigns not known; no control group
Canada			
Northern Antibiotic Resistance Partnership [21]	Cohort study	Reduction in MRSA infection rate and an increase in knowledge related to antimicrobial use and hand washing in the community	No data knowledge (adults and children) in nonintervention communities
Do Bugs Need Drugs program [22]	Uncontrolled interrupted time series	Program improved clinical knowledge and rate of appropriate antibiotic prescribing for upper respiratory tract infections. Ecological association between program implementation and stabilising of antibiotic prescribing and costs.	No control group
Greece			
A multifaceted campaign targeting both physicians and parents of school children on judicious use of antibiotics [23]	Uncontrolled before and after study	Overall antibiotic consumption was unchanged; however, the proportion of amoxicillin and phenoxymethylpenicillin used increased compared with a decrease in macrolides, cephalosporins, and fluoroquinolones	Seasonal and other temporal confounding factors not accounted for
Italy			
Toolkit for managing ESBL-E colonisation and infection [24]	Uncontrolled before and after study	Reduction in overall antibiotics prescribed from 60% of patients with asymptomatic ESBL-E to 39%	No control group
Sweden			
Strama [25]	Uncontrolled time series and institute publication	Reduction in outpatient antibiotic use, particularly in children aged 5–14 years and for macrolides	No control group
United Kingdom			
Enhanced AMS program in hospital and community [26]; Northern Ireland	Interrupted time series	Reduction in fluoroquinolone use and associated reduction in MRSA incidence in the community	No control group
Scottish Antimicrobial Prescribing Group [27]; Scotland	Descriptive study	Contributed to the reduction of *Clostridium difficile* infection rates, improved clinical management of infections	Nonexperimental study design
The Cornwall One Health Antimicrobial Resistance Group [28]	Descriptive study	Attributed reductions in antibiotic consumption by 12.8% in total (before and post-group formed) to the implementation of the TARGET toolkit (a national AMS toolkit for general practice)	Nonexperimental study design
Mixed persuasive and restrictive antibiotic stewardship intervention [29]; Scotland	Observational and quasiexperimental time-series analysis	Reducing population consumption of fiuoroquinolone, cephalosporins, clindamycin, and macrolides predicted large and sustained declines in *C*. *difficile* infection prevalence in both hospitals and the community. Associations with *C*. *difficile* infection occurred only where use of these antibiotics exceeded total use thresholds, consistent with the importance of selective pressures favouring epidemic ribotypes.	Further multicentre time-series analyses or cluster-randomised controlled trials would strengthen evidence
United States of America			
The Core Elements of Antibiotic Stewardship for Nursing Homes [30]	National guidance	Not evaluated	
A household- and office-based patient educational intervention and physician-centred intervention [31]	Controlled trial	Reduction in antibiotic prescription rate post-patient education and minor reduction in antibiotic prescription rate post-physician intervention	Claims data may miss emergency department data
Extending hospital-pharmacist–led AMS team services to hospital-affiliated nursing home [32]	Uncontrolled before and after study	Reduction in inappropriate antibiotic prescribing	
Introduction of an LID consult team (hospital infectious disease physician and nurse practitioner) to a long-term care facility [33]	Interrupted time-series study and cohort study	Reduced antibiotic use, particularly with tetracyclines, clindamycin sulfamethoxazole/trimethoprim, fluoroquinolones, and beta-lactam/beta-lactamase inhibitor combinations. Reduced positive *C*. *difficile* test rate.	Total days of therapy measured (not number of antimicrobial courses initiated)
Zambia			
BeatRHDZambia initiative[34]	Uncontrolled before and after study	Substantial changes in the pattern of benzathine penicillin G usage as a result of the intervention was reported but no data were presented	No control group

**Abbreviations**: AMS, antimicrobial stewardship; ESBL-E, extended-spectrum beta-lactamase producing Enterobacteriaceae; GP, general practitioner; LID, long-term care facility infectious disease; MRSA, methicillin-resistant *Staphylococcus aureus*; TARGET, Treat Antibiotics Responsibly, Guidance, Education, Tools.

**Table 3:** Stakeholders in the integrated AMS initiatives identified.

Study–AMS initiative	AMS Initiative Developed and Implemented by	Target Recipients for the AMS Initiative
Australia		
Infection control nurse consultant in residential aged care facilities [19]	GPs, infection control clinical nurse consultant, AMS team in residential aged care facility, and off-site hospital infectious disease physician	GPs in residential aged care facility
National multistrategic AMS program for health professionals and the community [20]	National Prescribing Service	GPs, community pharmacists, general public
Canada		
Northern Antibiotic Resistance Partnership [21]	University of Saskatchewan, Health Canada Research Ethics Boards	Primary healthcare providers, general public, school staff, and children
Do Bugs Need Drugs program [22]	Alberta Health Services (spanning primary and secondary care), Alberta Medical Association, University of Alberta, Alberta Lung Association, British Columbia Ministry of Health and British Columbia Centre for Disease Control. Healthcare providers and healthcare and early childhood education students were trained to deliver the public education sessions.	Children aged 2–5 and 7 years, their parents, older adults in assisted-living facilities, general public, community-based physicians and pharmacists
Greece		
A multifaceted campaign targeting both physicians and parents of school children on judicious use of antibiotics [23]	Medical school of the University of Athens, Prefecture of Corinth, Medical Association of Corinth, physician who specialised in infectious diseases	Primary care physicians, paediatricians, parents of children in nursing care and primary school, general public, dentists
Italy		
Toolkit for managing ESBL-E colonisation and infection [24]	An initiative led by a network of infectious diseases specialists in Southeastern France developed a warning system combined with a toolkit for managing ESBL-E colonisation or infection in collaboration with microbiologists from private laboratories and community-based GPs. The toolkit promoting French recommendations was implemented in Liguria, Italy (because there were no national recommendations at the time). This comprised a framework for establishing the warning system based on the availability of infectious diseases expert advice and the ESBL-E toolkit.	Prescribers in hospitals, elderly nursing homes, long-term care facilities, GPs
Sweden		
Strama [25]	Strama groups were established through the County Medical Officers for Communicable Diseases Control in every county. Groups had representatives from general practice and hospital (including general medicine, infectious diseases, paediatrics, otolaryngology, clinical microbiology, and infection control) and community pharmacies.	Broad audience including policy makers, physicians, and general public
United Kingdom		
Enhanced AMS program in hospital and community [26], Northern Ireland	General practice staff and hospital clinical staff	Hospital clinical staff, GPs
Scottish Antimicrobial Prescribing Group [27], Scotland	Hospital-based antimicrobial pharmacists, microbiologists, infectious disease specialists, hospital medical and nonmedical leadership, infection prevention specialists, information/antimicrobial surveillance scientists, GPs, dentistry, veterinary medicine, quality improvement, pharmaceutical industry, other expert advisors	Broad audience including policy makers, physicians, and general public
The Cornwall One Health Antimicrobial Resistance Group [28]	Developed by a subgroup of the Health & Wellbeing Board’s Health Protection Committee. The Chief Hospital Pharmacist and Medical Director initiated wide stakeholder engagement including members from wider hospital staff, clinical commissioning group, community hospital, out-of-hours GP service, dentistry, veterinary, and farming.	Broad audience including policy makers, physicians, and general public across sectors
Mixed persuasive and restrictive antibiotic stewardship intervention [29]; Scotland	Nationally developed but implemented by regional antimicrobial management teams.	Healthcare professionals in primary care, tertiary hospitals, district-general hospitals, and geriatric hospitals
United States of America		
The Core Elements of Antibiotic Stewardship for Nursing Homes [30]	Consultant pharmacist (community and/or hospital) and clinical and nursing staff	Nursing home staff
A household and office-based patient educational intervention and physician-centred intervention [31]	Colorado medical society and commercial and managed care organisation	Primary care physicians
Extending hospital-pharmacist–led AMS team services to hospital-affiliated nursing home [32]	Hospital internal medicine physician, pharmacists, infection control coordinator, and staff from nursing home	Prescribers in nursing home
LID consult team in a long-term care facility [33]	Hospital infectious disease physician and nurse practitioner and long-term care facility staff	Long-term care facility staff
Zambia		
BeatRHDZambia initiative [34]	Hospital microbiologists, infectious disease consultants, pharmacists, nurses, pharmaceutical advisors, GPs, academics, pharmaceutical advisors, representation from veterinary and farm services, representation from community pharmacy, Public Health England, representation from dental practice, public health educators, and public representation	General public, healthcare workers and vets, GPs, community pharmacies, urgent care centre staff, staff, and patients at the study hospital and government clinics in Lusaka

AMS initiatives, models, programs, and interventions are terms that are used interchangeably in the literature. Here, we use ‘AMS model’ to refer to a proposed simplified framework that outlines the structure, processes and intended outcomes associated with the goal of AMS [35]. Examples are the internationally recognised AMS model for hospitals from the Infectious Diseases Society of America and the Society for Healthcare Epidemiology of America [35,36] and British Society for Antimicrobial Chemotherapy [37]. An AMS intervention is any action taken with the aim of improving antimicrobial use, e.g., use of delayed/back-up antibiotic prescriptions or implementation of infection specialist approval for restricted antimicrobials. Accordingly, an AMS program describes a coordinated effort to improve antimicrobial use that involves two or more AMS interventions. **Abbreviations**: AMS, antimicrobial stewardship; ESBL-E, extended-spectrum beta-lactamase producing Enterobacteriaceae; GP, general practitioner; LID, long-term care facility infectious disease.

## Extent of AMS integration across the whole health economy

**Fig 1:**
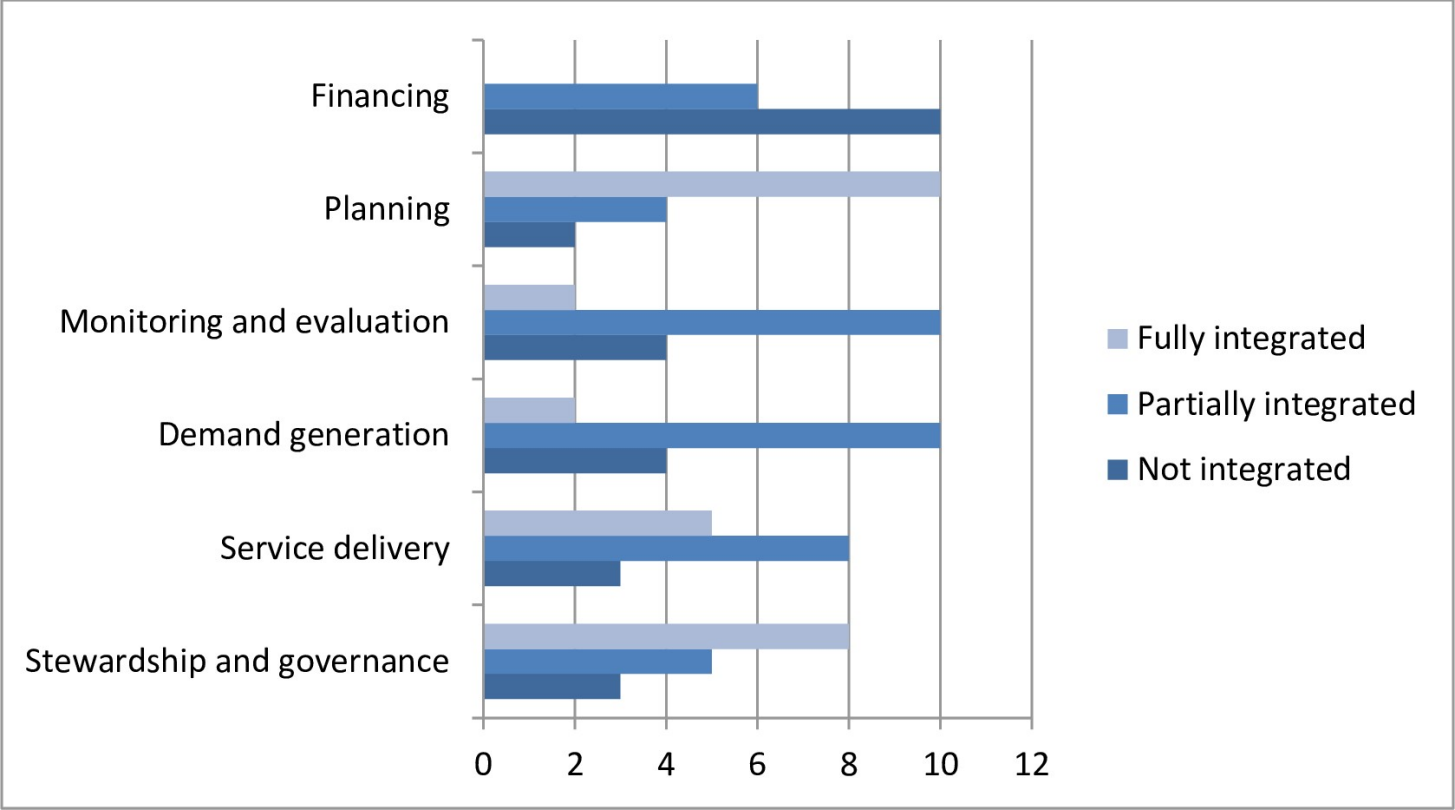
An overview of the extent of multisectoral AMS integration for each of the 16 AMS initiatives identified. The integration framework is based on all six facets of critical health system function defined by Atun and colleagues [16,18] (**Table 1**). AMS, antimicrobial stewardship.

Integration mapping of the 16 initiatives based on **Table 1** suggests that a range of approaches have been used to achieve multisectoral AMS (**Fig 1**). Full integration in Planning was often considered a key factor for establishing many initiatives coupled with an integrated Stewardship and Governance approach. Integration in these two facets was mainly achieved through expansion of the AMS program, by which the primary governance responsibilities remained with the host institution [19,20,30,32,33], rather than through establishment of new structures [25]. AMS initiatives that had a shared governance structure across healthcare organisations (i.e., partially integrated) were either national programs [27] or state-wide programs [26,31]. While these provide examples of an integrated AMS governance approach, effective governance is likely to require much more than a multistakeholder approach to plan and deliver services; a mixed regulatory and persuasive strategy including effective public engagement is needed [38]. In our analysis, nine initiatives were partially integrated for Demand Generation, showing a potential missed opportunity for this critical facet that includes raising awareness and increasing engagement with the public, practitioners, health service managers, and policymakers. Monitoring and Evaluation relate to the functions around data collection, analysis, reporting, and performance-management systems. Full integration was identified in one initiative in which the health system oversaw these functions regionally or was responsible for these functions directly [33]. More often, data collection and analyses were managed by the wider health system; however, performance management roles were not [19,20,22,25,26,32]. Financing relates to the pooling of funds/funding source, cross-program use of funds, and provider payment methods involved in the AMS initiative. The majority of initiatives did not report on how they were or should be financed or how the funds were or should be used [19,20,22,23,26,30,32]. While fund pooling was partially integrated in three initiatives [25,32,33], decisions for provider payment methods were not. Overall, 11 studies evaluated the AMS initiative using mainly quasiexperimental study designs [19–23,25,27,30,31,39] (**S2 Table**). These reported on a range of positive impacts including reductions in antibiotic prescribing, reductions in the proportion of broad-spectrum antibiotic prescribed, reduction in *C*. *difficile* infection rates, and perceived improvement in citizens’ knowledge and attitudes about self-management of minor infections. However, potential for bias should be borne in mind because of study limitations associated with uncontrolled research designs, insufficient data time points, and risk of self-selection by participants who are interested in AMS.

## Opportunities and implications for policy

Especially when planning new initiatives, a health system function framework as employed here can be critical to minimise duplication of effort and achieve efficiencies from the viewpoint of healthcare professionals and service users. Our assessment has highlighted strengths of initiatives associated with beneficial outcomes, and we present these as three interconnected practical recommendations for policymakers to consider.

### A successful integrated AMS approach can be developed through expansion of an existing AMS program

When compared to hospital-based AMS, strategies within primary care and long-term care have generally been slow to develop. Outside of hospitals, structural constraints sometimes include undefined AMS leadership at the organisational level and therefore unclear responsibilities around local AMS objectives and lack of timely pathways to specialist support. An integrated AMS model, particularly one involving secondary care, can overcome some of the community-based issues by either extending existing secondary-care AMS programs [19,26,32,33], adapting from established frameworks for secondary-care AMS [30], or creating a joint platform for multisectoral AMS strategies to be presented, developed, monitored, and/or shared [25,27]. It therefore follows that an integrated AMS program may also be able to address process issues such as fragmented and timely follow-up of patients, their symptom progression, and medical management. However, further research is required to investigate this. Critically, there is a need for establishing sustainable funding for AMS teams working beyond hospital settings that is not solely derived from cost savings through reduced drug expenditure. Instead, funding for developing and supporting AMS teams should be considered within the patient safety and healthcare-quality–related spending [40]. Irrespective of these issues, adoption and uptake of AMS strategies are likely to be influenced by the underlying health system and culture in a country.

### Opportunities for success establishing consistent communication channels with responsibilities and common goals clearly defined

Few health systems appear to have effective mechanisms for sharing and disseminating learning about AMS, leading to small-scale local initiatives. Strengthening communication between commissioners, providers, and consumers by having more structured and clear communication pathways, such as in the Strama model developed in Sweden and the similarly structured Scottish Antimicrobial Prescribing Group, can be an effective way to develop, disseminate, and monitor ways to improve AMS [25,27].

### Capitalise on existing resources and processes

Patients and the public have a pivotal role in infection prevention and management, yet failure to involve and engage with them in decision-making or achieve sustained behaviour change remains a problem in all health sector settings [41,42]. We found few examples of patient or public involvement in the design and delivery of integrated AMS initiatives (**Table 3**). However, we know from other studies that patient misconceptions about AMR and what constitutes appropriate antibiotic use is a major driver for inappropriate behaviours around antibiotic use [43]. Furthermore, our stakeholder analysis suggests that there are potentially more opportunities for integration, particularly involving primary care service providers. For instance, in the United Kingdom, it is well recognised that nurses and pharmacists in the community are generally more accessible to the public than general practitioners (GPs). The continuing expansion of their roles in the community, which not only provides support to patients but also reduces the burden on primary care physicians, is testament to this [44,45]. However, there are few AMS initiatives that capitalise on these valuable resources to deliver integrated AMS—by this, we mean appropriate antibiotic access and preservation and knowledge mobilisation for promoting AMS that is aligned with primary, secondary, tertiary, and long-term institutional care sectors. We found little involvement of dental practitioners in most multisectoral AMS initiatives, which is another missed opportunity. Further work is required to investigate such AMS roles in the community and embed these more widely as applicable in the respective country. A more robust evidence base is needed to establish the effectiveness of integrated AMS initiatives and specifically consider contextual antecedents to better inform future sustained improvements.

Overall, we urge policymakers, clinical leaders, and healthcare managers to assess and consider consolidating AMS activities across the whole health economy. Each of these stakeholders have an important role to drive and support clinicians, researchers, and research-active patients to carry out quality research that will inform the development of more robust evidence-based policies and guidelines. The analytic framework presented here can be used to assess the extent of integration of existing or planned multisectoral AMS initiatives, and we have outlined three areas with practical considerations towards how future integration of AMS initiatives across the whole health economy may be achieved. Ultimately, integrated AMS must prove itself as an essential element of efficient redesign if it is to deliver sustained patient benefits.

## Supporting information

S1 Appendix Search strategy and article selection. (PDF)

S1 Table Frameworks for assessing extent of integration considered. (PDF)

S2 Table Overview of 15 integrated AMS initiatives. AMS, antimicrobial stewardship.
(PDF)

## Abbreviations

AMR: antimicrobial resistance
AMS: antimicrobial stewardship
ESBL-E: extended-spectrum beta-lactamase producing Enterobacteriaceae
GP: general practitioner
LID: long-term care facility infectious disease
MRSA: methicillin-resistant *Staphylococcus aureus*
NHS: National Health Service
NIHR: National Institute for Health Research
PHE: Public Health England
TARGET: Treat Antibiotics Responsibly, Guidance, Education, Tools.
