# Supplementary material for: A whole-health–economy approach to antimicrobial stewardship: Analysis of current models and future direction
Source: PLoS Med. 2019 Mar 29;16(3):e1002774. doi: 10.1371/journal.pmed.1002774 (PMC6440619; doi:10.1371/journal.pmed.1002774)
Supplement: S1 Appendix — (PDF) [file pmed.1002774.s001.pdf]

S1 Appendix. SEARCH STRATEGY AND ARTICLE SELECTION

Search strategy

Seven electronic databases (EMBASE, Medline, Cochrane library, Cochrane Clinical Trials, NHS EED, Cochrane Health Technology Assessment, Cochrane other reviews) were searched from January 2006 to December 2018. We searched for the following in the title and abstracts: free-text "antimicrobial" and "stewardship", their associated synonyms and database-specific thesaurus terms (e.g. EMBASE search terms were ANTI-INFECTIVE AGENT/ OR ("antimicrobial"[ti.ab]) OR ("antibiotic"[ti.ab]) OR ("anti-infective"[ti.ab]) OR ("anti-microbial"[ti.ab]) OR ("anti-biotic"[ti.ab], ("stewardship"[ti.ab]) OR HEALTH PROGRAM/ OR HEALTH PROGRAMME/ OR HEALTH PROMOTING BEHAVIOR/ OR HEALTH PROMOTING BEHAVIOUR/ OR HEALTH PROMOTION/ OR HEALTH PROMOTION MODEL/). We also searched 25 websites of healthcare institutions that provide peer-reviewed guidance and/or information pertaining to designing and implementing AMS initiatives[1]. Studies included in recent literature reviews and reference lists of full text articles were scrutinised for inclusion eligibility [2,3].

Selection process and data extraction

Each title and abstract was reviewed independently by two researchers (RA,MS,NS,CM). All articles describing an AMS intervention, programme or model were included if either: (1) vertical integration was apparent (Fig A), i.e. more than one healthcare organisation in ANY COMBINATION of primary care, secondary care, tertiary care and/or long-term healthcare institutional care; (2) horizontal integration was apparent i.e. more than one healthcare organisation WITHIN ONE SECTOR from primary care, secondary care, tertiary care and/or long-term healthcare institutional care; OR (3) AMS was targeted at staff or patients/public in two or more healthcare sectors. A summary of the selection process is shown in Fig B.

Two researchers [from MM, RA, CM], extracted data and carried out quality assessment independently using a pre-piloted form. Discrepancies were resolved via a third researcher [MM, RA, CM, NS]. Quality assessment of included articles was based on nine questions adapted from two frameworks as no single quality assessment criteria framework was sufficient for the included range of articles and study designs [4,5] : (1) Was the aim of the AS model/programme/intervention clear? (2) Was the AS model/programme/intervention clearly described? (3) Were the stakeholders the AS model/programme/intervention is aimed at identified (4) Were personnel from multiple disciplines involved in delivering the AS model/programme/intervention specified? (5) Was the span (organisations, institutions, geographic location) of the integrated AS model/program/intervention specified? (6) Were outcome measures for monitoring and evaluating the AS model/programme/intervention identified? (7) Has the AS model/programme/intervention been evaluated? (8) Was the sustainability of the AS model/programme/intervention considered? (9) Were key limitations of the AS model/programme/intervention considered? The quality assessment was used to assess the strengths and limitations of studies, and not as an exclusion criterion.

Fig A. Schematic of horizontal and vertical multi-sectoral health care integration.

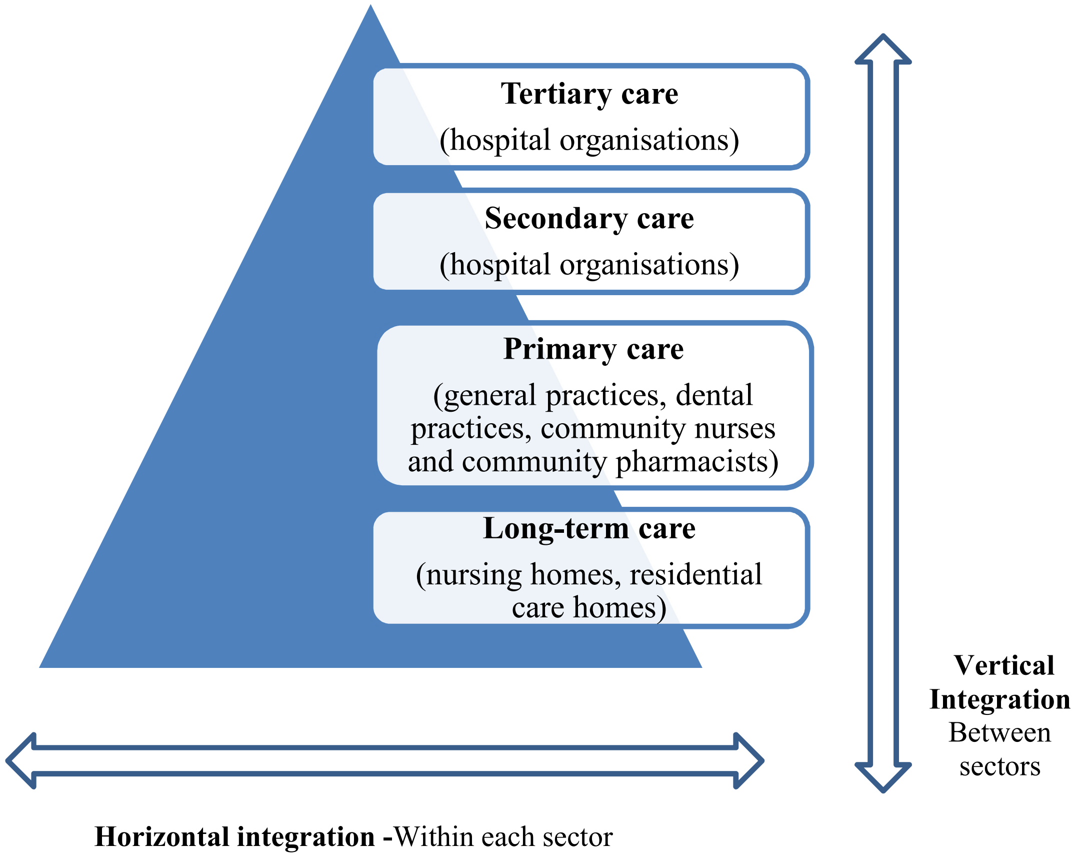

Fig B. Flow diagram of study selection. Abbreviations: AMS, antimicrobial stewardship

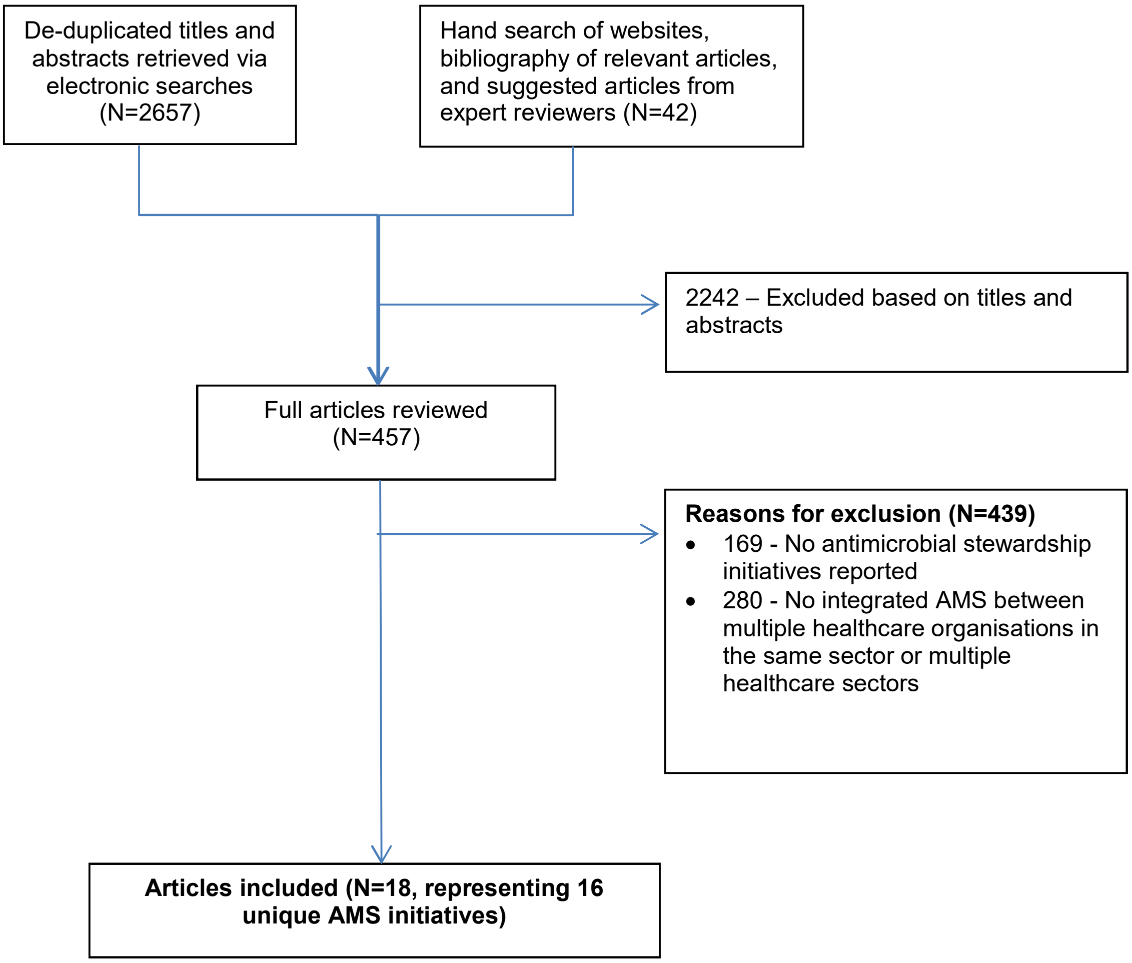

References

1. Pagani L, Gyssens IC, Huttner B, Nathwani D, Harbarth S. Navigating the Web in search of resources on antimicrobial stewardship in health care institutions. Clin Infect Dis. 2009;48: 626–632. doi:10.1086/596762
2. Davey P, Marwick C, Scott C, Charani E, McNeil K, Brown E, et al. Interventions to improve antibiotic prescribing practices for hospital inpatients (Review). Cochrane database Syst Rev. 2017; CD003543. doi:10.1002/14651858.CD003543.pub4.www.cochranelibrary.com
3. Patel SJ, Larson EL, Kubin CJ, Saiman L. A review of antimicrobial control strategies in hospitalized and ambulatory pediatric populations. Pediatr Infect Dis J. 2007;26: 531–7. doi:10.1097/INF.0b013e3180593170
4. Zingg W, Castro-Sánchez E, Secci F V., Edwards R, Drumright LN, Sevdalis N, et al. Innovative tools for quality assessment: Integrated quality criteria for review of multiple study designs (ICROMS). Public Health. Elsevier Ltd; 2016;133: 19–37. doi:10.1016/j.puhe.2015.10.012
5. Dellit TH, Owens RC, McGowan JE, Gerding DN, Weinstein R a, Burke JP, et al. Infectious Diseases Society of America and the Society for Healthcare Epidemiology of America guidelines for developing an institutional program to enhance antimicrobial stewardship. Clin Infect Dis. 2007;44: 159–77. doi:10.1086/510393
