# Supplementary material for: A whole-health–economy approach to antimicrobial stewardship: Analysis of current models and future direction
Source: PLoS Med. 2019 Mar 29;16(3):e1002774. doi: 10.1371/journal.pmed.1002774 (PMC6440619; doi:10.1371/journal.pmed.1002774)
Supplement: S1 Table — (PDF) [file pmed.1002774.s002.pdf]

S1 Table. Frameworks for assessing extent of integration considered.

Model	Major components
Valentijin, 2017 [1]	Clinical integration<br>Functional integration<br>Normative integration<br>Organisational integration<br>Professional integration<br>System integration<br>Assessed for: Population based care, Person-focused care, Population based care<br>At levels: micro, meso, macro
Atun et al, 2010 [2]	Stewardship and governance<br>Financing<br>Planning<br>Service delivery<br>Monitoring and evaluation<br>Demand generation
Curry and Ham, 2010 [3]	Level of integration: Macro; Meso; Micro
Fulop et al, 2005 [4]	Clinical integration<br>Functional integration<br>Normative integration<br>Organisational integration<br>Service integration<br>Systemic integration
Contandriopoulos et al, 2003 [5]	Care integration<br>Clinical team<br>Functional integration<br>Normative integration<br>Systemic integration
Shortell, 2000 [6]	Clinical integration - micro level (continuity, co-operation and coherence in the primary process of care delivery to individual patients)<br>Functional integration - macro level (financing, regulation)<br>Organisational integration - meso level (mergers, contracting or strategic alliances)<br>Professional integration - meso level (mergers, strategic alliances between health care professionals).

References

1. Valentijn PP, Schepman SM, Opheij W, Bruijnzeels MA. Understanding integrated care: a comprehensive conceptual framework based on the integrative functions of primary care. Int J Integr Care. Ubiquity Press; 2013;13. doi:10.5334/ijic.886
2. Atun R, De Jongh T, Secci F, Ohiri K, Adeyi O. Integration of targeted health interventions into health systems: A conceptual framework for analysis. Health Policy Plan. 2010;25: 104–111. doi:10.1093/heapol/czp055
3. Curry N, Ham C. Clinical and service integration: the route to improved outcomes [Internet]. London; 2010. Available: www.kingsfund.org.uk/publications
4. The NHS Confederation. Building integrated care. Lessons from the UK and elsewhere. London: The NHS Confederation; 2005.
5. Contandriopoulos A-P, Denis J-L, Touati N, Rodríguez C. The integration of health care: dimensions and implementation. Montreal: Groupe de recherche interdisciplinaire en sante; 2003.
6. Shortell SM (Stephen M. Remaking health care in America : building organized delivery systems [Internet]. Jossey-Bass Publishers; 2000.
