# Supplementary material for: A whole-health–economy approach to antimicrobial stewardship: Analysis of current models and future direction
Source: PLoS Med. 2019 Mar 29;16(3):e1002774. doi: 10.1371/journal.pmed.1002774 (PMC6440619; doi:10.1371/journal.pmed.1002774)
Supplement: S2 Table — AMS, antimicrobial stewardship. (PDF) [file pmed.1002774.s003.pdf]

S2 Table. Overview of 16 integrated antimicrobial stewardship (AMS) initiatives.

Study – AMS initiative	Aim/context	Study design	Description
Australia			
Infection control nurse consultant in residential aged care facilities [1]	To assess the role of the infection control clinical nurse consultant in the AMS team in two residential aged care facilities	Uncontrolled before and after study	**AMS overview**: Implementation of an infection control clinical nurse consultant in the AMS team in two residential aged care facilities. Activities include lectures to nursing staff and GPs, audit and feedback, reminders on appropriate prescribing, and monitoring of pathology results.<br>**Multi-sectoral AMS components**: The nurse consultant acted as an intermediary between the prescribing GP and an off-site infectious diseases physician,<br>**Reported impact**: Reduction in the use of cephalexin, doxycycline, flucloxacillin, clindamycin and metronidazole. Rates of infection types remained stable except respiratory tract infection rates increased at one of the two study sites.
National multi-strategic AMS programme for health professionals and the community[2]	To evaluate a national programme aimed at reducing inappropriate use of antibiotics for upper respiratory tract infections	Uncontrolled before and after study series	**AMS overview**: The National Prescribing Service Ltd undertook a multi-strategic AMS programme for HCPs and the community.<br>**Multi -sectoral AMS components**: Interventions were targeted at HCPs and the public/consumer which included creation of a tagline "Common colds need common sense" and adapted in subsequent years, community awareness campaign comprised radio and television interviews, newspaper articles, and advertising on billboards, bus shelters and at railway stations. Printed and electronic resources were distributed including information brochures and posters for general practice, pharmacies, schools, and community centres. GPs were provided with prescription pads for symptomatic management and patient information leaflets on sore throat and cough were distributed and included in some prescribing software. Small grants (approx. $AUD300-600) were provided to community groups including child-care groups to implement community based education<br>**Reported impact:** Continued decline in total volume of antibiotics prescribed, GPs and pharmacists perceived the campaign assisted in AMS message promotion to patients, improvement in consumer knowledge and attitudes about self-management of infections.
Canada			
Northern Antibiotic Resistance Partnership[3]	To develop physician, patient, community, and school based educational materials to limit the spread of community-acquired MRSA	Cohort	**AMS overview**: Formation of the Northern Antibiotic Resistance Partnership comprising community members, HCPs, educators and research scientists to develop, monitor and coordinate AMS strategy.<br>**Multi -sectoral AMS items**: Multi-faceted phased programme of interventions targeting stakeholders in multiple sectors, involving: educational activities, active surveillance system to enhance data collection on microbiological sensitivities, prescribing practices and general patient demographic data, information to physicians and healthcare providers on MRSA infection risk factors, local guidelines, posters, radio interviews and slide presentations were provided to targeted communities, training sessions was rolled-out to children in targeted communities and provision of pocket chart.<br>**Reported impact**: Reduction in MRSA infection rate and increase in knowledge related to antimicrobial use and hand washing in the community.
Do Bugs Need Drugs program[4]	To evaluate the impact of the Do Bugs Need Drugs educational program	Uncontrolled interrupted time series	**AMS overview:** Implementation of the Do Bugs Need Drugs program that aims to reduce the number of unnecessary antibiotic prescriptions.<br>**Multi -sectoral AMS items**: Interventions were focussed on educating the public and HCPs on the appropriate use of antibiotics. Interventions included annual media campaigns, print material distribution, and education sessions. HCP education included: provision of accredited courses to physicians and pharmacists, associated assessments, and trained a number of healthcare provider and early childhood education students to deliver the public education sessions.<br>**Reported impact**: Programme improved clinical knowledge and rate of appropriate antibiotic prescribing for URTI
Greece			
A multifaceted campaign targeting both physicians and parents of school children on judicious use of antibiotics [5]	To evaluate the impact of a multifaceted educational campaign targeting physicians and parents of school	Uncontrolled before and after study	**AMS overview**: multifaceted campaign targeting both physicians and parents of school children on judicious use of antibiotics.<br>**Multi -sectoral AMS items**: 2-hour education meetings with parents of children in nursing care and primary school, lecture from infectious disease physician, educational pamphlet on the use of antibiotics for common infections published by the Institute of Pharmaceutical Research and Technology, press-conference at the start of the campaign. Academic detailing were targeted at GPs, paediatricians, otorhinolaryngologists, chest physician delivered via lectures and an interactive session, provision of management guidelines for community acquired infections.

Study – AMS initiative	Aim/context	Study design	Description
	children on judicious use of antibiotics		**Reported impact**: Overall antibiotic consumption was unchanged, however the proportion of amoxicillin and phenoxymethylpenicillin used increased compared with a decrease in macrolides, cephalosporins and fluoroquinolones.
Italy			
Toolkit for managing extended-spectrum beta-lactamase producing Enterobacteriaceae infection and colonization [6]	To report on the outcome of an intervention aiming to promote antimicrobial stewardship through organized management of extended-spectrum beta-lactamase producing Enterobacteriaceae infection and colonization	Uncontrolled before and after study	**AMS overview:** As an initiative led by a network of infectious diseases specialists in Southeastern France (Réso-Infectio-PACA-Est) (http://www.reso-infectio.fr/) including all local public hospitals and several private clinics in the Eastern part of the Provence Alpes Côte d'Azur region, a warning system combined with a toolkit for managing extended-spectrum beta-lactamase producing Enterobacteriaceae colonization or infection was developed in collaboration with microbiologists from private laboratories and community-based general practitioners. **Multi-sectoral AMS items:** As part of an EU-sponsored cross-border cooperation (http://www.interreg-alcotra.org/2007-2013/index.php?pg=progetto&id=231), the proposition consisted in offering a framework in order to establish a warning system, based on the availability of infectious diseases expert advice, and providing the extended-spectrum beta-lactamase producing Enterobacteriaceae toolkit, once customized for antimicrobial agents available in Italy, to healthcare professionals in an administrative district within Liguria. This area includes a population of 214,000, 3 hospitals, 4 elderly nursing homes, 31 long-term care facilities, and 180 GPs. **Reported impact:** Reduction in overall antibiotics prescribed from 60% of patients with asymptomatic ESBL-E to 39%.
Sweden			
STRAMA [7]	To describe the STRAMA programme and summarise the results of the first 10 years	Uncontrolled time series and Institute publication	**AMS overview**: STRAMA comprise a voluntary network at two levels: national and local. National activities: monitoring AMR and antibiotic use, revision of national treatment recommendations, developing a repository of training material, monitor international developments, survey antibiotic prescribing, communicating the issue of AMR to HCPs, the media, the general public and decision-makers. Local activities: monitoring resistance and prescribing patterns, participating in annual point prevalence studies on healthcare associated infections, deciding on annual activity plans and targets, regular visits and feedback to general practitioners and hospital prescribers, local adaptation of national treatment guidelines, training of HCPs, and a local awareness campaigns targeting the general public **Multi -sectoral AMS items**: STRAMA is the platform for gathering stakeholders for knowledge exchange, identifying needs and implementation of treatment guidelines/policies. **Reported impact**: Reduction in outpatient antibiotic use, particularly in children aged 5-14 years and for macrolides.
United Kingdom			
Enhanced AMS programme in hospital and community [8]	To evaluate the impact of an AMS programme on MRSA rate in primary and secondary healthcare settings	Interrupted time series	**AMS overview**: enhanced AMS programme in hospital and community. **Multi -sectoral AMS items**: Hospital-led AMS intervention comprising information leaflet, quarterly prescribing feedback to GPs, and training on appropriate antibiotic use to GPs. **Reported impact**: Reduction in fluoroquinolone use and associated reduction in MRSA incidence in the community.
Scottish Antimicrobial Prescribing Group [9]	To coordinate and deliver a national programme of work for AMS	Descriptive	**AMS overview**: the Scottish Antimicrobial Prescribing Group was established in 2008 and aims to co-ordinate and deliver a national programme of work for AMS. Some of this work is embedded in the Scottish Patient Safety Programme. **Multi -sectoral AMS items**: AMS interventions were delivered locally through NHS board antimicrobial management teams. There are five workstreams, all of which involve both vertical and horizontal integration and is led by an expert in the specific area. A set of prescribing indicators for primary care and hospitals were also developed. AMS interventions include: development of national guidance; development of systems for the collection, analysis and reporting of information relating to antimicrobial prescribing and resistance in all healthcare settings; developing training materials for undergraduate and postgraduate HCPs including a framework of learning outcomes for AMS; elearning resources for HCPs; training pharmacists in community and hospital; integrating antimicrobial recommendations in surgery into the Scottish Patient Safety Programme checklist PAUSE. **Reported impact**: Contributed to the reduction of *Clostridium difficile* infection rates, improved clinical management of infections.

Study – AMS initiative	Aim/context	Study design	Description
The Cornwall One Health Antimicrobial Resistance Group[10]	To describe the inauguration of the Cornwall Antimicrobial Resistance Group and associated outputs	Descriptive study	**AMS overview:** the Cornwall Antimicrobial Resistance Group was set up in 2014 with the purpose of implementing a coordinated Cornwall-wide response to the UK Strategy for tackling Antimicrobial Resistance. Specifically, the group aimed to: (i) improve professional eduction, training and public engagement; (ii) optimize prescribing practice; (iii) improve access and use of surveillance data.<br>**Multi-sectoral AMS items:** The group comprised stakeholder organizations from primary care commissioning, secondary care service provider, community hospital, out of hours general practice service, community pharmacy, veterinary and farm services, Public Health England, and academia.<br>**Reported impact:** Attributed reductions in antibiotic consumption by 12.8% in total (before and post group formed) to the implementation of the TARGET toolkit (a national AMS toolkit for general practice)
Mixed persuasive and restrictive antibiotic stewardship intervention[11]	To assess the effect of antibiotic stewardship and infection prevention and control on the clinical and molecular epidemiology of *Clostridium difficile* infection in hospitals and community populations of a Scottish health board.	Observational and quasi-experimental time-series analysis	**AMS overview:** persuasive elements included empirical antibiotic therapy guidelines avoiding use of 4C antibiotics (ciprofloxacin/fluroquinolones, co-amoxiclav, clindamycin, and cephalosporins), reminders for clinical staff , and feedback on prescribing, through root cause analysis for all episodes of *Clostridium difficile* infection and ward-based auditing. Restrictive aspects included removal of 4C antibiotic stocks from wards, use of these drugs requiring authorisation from a medical microbiologist, and non-disclosure of sensitivities to 4C antibiotics in microbiology reports, unless there were no alternatives.<br>**Multi-sectoral AMS items:** A regional antibiotic management team, comprised of primary care and antibiotic pharmacists, and microbiology and infectious disease specialists, led implementation. National reports comparing performance of health boards against prescribing quality indicators were made public. Funding for the antibiotic stewardship intervention was provided by NHS Scotland.<br>**Reported impact:** Reducing population consumption of fluoroquinolone, cephalosporins, clindamycin, and macrolides predicted large and sustained declines in *Clostridium difficile* infection prevalence in both hospitals and the community. Associations with *Clostridium difficile* infection occurred only where use of these antibiotics exceeded total use thresholds, consistent with the importance of selective pressures favouring epidemic ribotypes.
United States of America			
The Core Elements of Antibiotic Stewardship for Nursing Homes[12]	Adaptation of existing hospital AMS recommendations for nursing homes	Review & Recommendations	**AMS overview**: the Core Elements of Antibiotic Stewardship for Nursing Homes.<br>**Multi -sectoral AMS items**: Establish multidisciplinary engagement both internal and external to nursing home on core elements: leadership, accountability, drug expertise, actions, tracking, reporting and education. Linking nursing home staff with infection prevention coordinator, laboratory services, state and local health departments, AMS leads in hospitals within referral network, infectious disease consultants in the community.<br>**Reported impact:** Not applicable
A household and office-based patient educational intervention and physician-centred intervention[13]	To assess the impact of patient education on antibiotic prescribing to children with pharyngitis and adults with acute bronchitis in private office practices	Controlled trial	**AMS overview**: A household and office-based patient educational intervention and physician-centred intervention<br>**Multi -sectoral AMS items**: Patient education comprised antibiotic resistance brochure, refrigerator magnet and a fact card about acute respiratory tract infections. Physician intervention comprised individual feedback of prescribing profile and practice guidelines.<br>**Reported impact:** Reduction in antibiotic prescription rate post patient education and relatively minor reduction in antibiotic prescription rate post physician intervention.
Extending hospital pharmacist-led AMS team services to hospital affiliated nursing home[14]	To determine whether pharmacists could improve antibiotic prescribing in a nursing home.	Uncontrolled before and after study	**AMS overview**: Intervention comprised extending hospital pharmacist-led AMS team services to hospital-affiliated nursing home.<br>**Multi -sectoral AMS items**: Hospital based team reviewed patients in nursing home who had cultures collected.<br>**Reported impact:** Reduction in inappropriate antibiotic prescribing
Introduction of a long term care facility infectious disease	To determine the impact of the long-term care facility	Interrupted time series study and cohort study	**AMS overview**: Introduction of a long term care facility infectious disease consult (LID) team (hospital infectious disease physician and nurse practitioner) to a long term care facility.<br>**Multi -sectoral AMS items**: LID team examined residents at the long term care facility weekly and were available for remote

Study – AMS initiative	Aim/context	Study design	Description
consult team (hospital infectious disease physician and nurse practitioner) to a long term care facility [15]	infectious disease consult service on antimicrobial use and *Clostridium difficile* infections		consultation the remainder of the week.<br>**Reported impact**: Reduced antibiotic use, particularly with tetracyclines, clindamycin sulfamethoxazole/trimethoprim, fluoroquinolones and beta-lactam/beta-lactamase inhibitor combinations. Reduced positive *Clostridium difficile* test rate.
Zambia			
Multi-faceted initiative (called ‘BeatRHD Zambia’) [16]	To describe and report initial signs of success of the BeatRHD Zambia programme on appropriate use of benzathine penicillin.	Uncontrolled before and after study	**AMS overview:** In 2012, a public–private partnership was launched in Zambia with the goal of reducing and ultimately eliminating rheumatic heart disease.<br>**Multi-sectoral AMS items:** This multi-faceted initiative (called ‘BeatRHD Zambia’) is centred out of the University Teaching Hospital (UTH) in Lusaka, Zambia, and includes operational research (for example, to measure disease prevalence), public awareness, and health system-strengthening activities – in particular, efforts to increase appropriate benzathine penicillin G usage for primary and secondary prevention of rheumatic heart disease in government health facilities according to national guidelines.<br>**Reported impact**: Substantial changes in the pattern of benzathine penicillin G usage as a result of the intervention was reported but (no data were presented)

Abbreviations: AMR, antimicrobial resistance; AMS, antimicrobial stewardship; GP, general practitioner; HCP, healthcare professional; MRSA, methicillin-resistant *Staphylococcus aureus;* URTI, upper respiratory tract infection

References:

1. Stuart RL, Orr E, Kotsanas D, Gillespie EE. A nurse-led antimicrobial stewardship intervention in two residential aged care facilities. Healthc Infect. 2015;20: 4–6. doi:http://dx.doi.org/10.1071/HI14016
2. Wutzke SE, Artist MA, Kehoe LA, Fletcher M, Mackson JM, Weekes LM. Evaluation of a national programme to reduce inappropriate use of antibiotics for upper respiratory tract infections : effects on consumer awareness , beliefs , attitudes and behaviour in Australia. Health Promot Int. 2007;22: 53–64.
3. Golding GR, Quinn B, Bergstrom K, Stockdale D, Woods S, Nsungu M, et al. Community-based educational intervention to limit the dissemination of community-associated methicillin-resistant Staphylococcus aureus in Northern Saskatchewan, Canada. BMC Public Health. 2012;12: 15.
4. McKay RM, Vrbova L, Fuertes E, Chong M, David S, Dreher K, et al. Evaluation of the do bugs need drugs? Program in British Columbia: Can we curb antibiotic prescribing? Can J Infect Dis Med Microbiol. 2011;22: 19–24.
5. Plachouras D, Antoniadou A, Giannitsioti E, Galani L, Katsarolis I, Kavatha D, et al. Promoting prudent use of antibiotics: the experience from a multifaceted regional campaign in Greece. BMC Public Health. 2014;14: 866. doi:10.1186/1471-2458-14-866
6. Mondain V, Secondo G, Guttmann R, Ferrea G, Dusi A, Giacomini M, et al. A toolkit for the management of infection or colonization by extended-spectrum beta-lactamase producing Enterobacteriaceae in Italy: implementation and outcome of a European project. Eur J Clin Microbiol Infect Dis. Springer Berlin Heidelberg; 2018;37: 987–992. doi:10.1007/s10096-018-3202-1
7. Mölstad S, Erntell M, Hanberger H, Melander E, Norman C, Skoog G, et al. Sustained reduction of antibiotic use and low bacterial resistance: 10-year follow-up of the Swedish Strama programme. Lancet Infect Dis. 2008;8: 125–132. doi:10.1016/S1473-3099(08)70017-3
8. Aldeyab MA, Scott MG, Kearney MP, Alahmadi YM, Magee FA, Conlon G, et al. Impact of an enhanced antibiotic stewardship on reducing methicillin-resistant Staphylococcus aureus in primary and secondary healthcare settings. Epidemiol Infect. 2014;142: 494–500. doi:10.1017/S0950268813001374
9. Nathwani D, Sneddon J, Malcolm W, Wiuff C, Patton A, Hurding S, et al. Scottish Antimicrobial Prescribing Group (SAPG): Development and impact of the Scottish National Antimicrobial Stewardship Programme. Int J Antimicrob Agents. 2011;38: 16–26. doi:10.1016/j.ijantimicag.2011.02.005
10. Powell N, Davidson I, Yelling P, Collinson A, Pollard A, Johnson L, et al. Developing a local antimicrobial resistance action plan: the Cornwall One Health Antimicrobial Resistance Group. J Antimicrob Chemother. 2017;72: 2661–2665. doi:10.1093/jac/dkx164
11. Lawes T, Lopez-Lozano J-M, Nebot CA, Macartney G, Subbarao-Sharma R, Wares KD, et al. Effect of a national 4C antibiotic stewardship intervention on the clinical and molecular epidemiology of Clostridium difficile infections in a region of Scotland: a non-linear time-series analysis. Lancet Infect Dis. 2017;17: 194–206. doi:10.1016/S1473-3099(16)30397-8
12. Centers for Disease Control and Prevention. The Core Elements of Antibiotic Stewardship for Nursing Homes [Internet]. Atlanta: US Department of Health and Human Services; 2015. Available: http://www.cdc.gov/longtermcare/prevention/antibiotic-stewardship.html
13. Gonzales R, Corbett KK, Leeman-Castillo BA, Glazner J, Erbacher K, Darr CA, et al. The ‘“ Minimizing Antibiotic Resistance in Colorado ”’ Project : Impact of Patient Education in Improving Antibiotic Use in Private Office Practices. Health Serv Res. 2005;40: 101–116.

14. Gugkaeva Z, Franson M. Pharmacist-led model of antibiotic stewardship in a long-term care facility. Ann Long-Term Care. 2012;20: 22–26.
15. Jump RLP, Olds DM, Seifi N, Kypriotakis G, Jury LA, Peron EP, et al. Effective antimicrobial stewardship in a long-term care facility through an infectious disease consultation service: keeping a LID on antibiotic use. Infect Control Hosp Epidemiol. 2012;33: 1185–1192. doi:10.1086/668429
16. Long A, Lungu JC, Machila E, Schwaninger S, Spector J, Tadmor B, et al. A programme to increase appropriate usage of benzathine penicillin for management of streptococcal pharyngitis and rheumatic heart disease in Zambia. Cardiovasc J Afr. 2017;28: 242–247. doi:10.5830/CVJA-2017-002
